# Gene variants of glucocorticoid activation pathways and the outcomes of patients with Takayasu arteritis – a retrospective cohort study

**DOI:** 10.3389/fimmu.2025.1675026

**Published:** 2025-10-02

**Authors:** Faustino Peron Filho, Andressa de Souza Moreira, Eduarda Bonelli Zarur, Gerson Dierley Keppeke, Alexandre Wagner Silva de Souza

**Affiliations:** ^1^ Rheumatology Division, Department of Medicine, Escola Paulista de Medicina, Universidade Federal de São Paulo, São Paulo, Brazil; ^2^ Rheumatology Division, Universidade do Estado do Rio de Janeiro, Rio de Janeiro, Brazil; ^3^ Departamento de Ciencias Biomédicas, Facultad de Medicina, Universidad Católica del Norte, Coquimbo, Chile

**Keywords:** Takayasu arteritis, glucocorticoids, drug toxicity, single nucleotide polymorphism, genetic studies

## Abstract

**Objective:**

This study aims to evaluate the influence of polymorphisms of the *HSD11B1, FKBP5* and *NR3C1* genes on the outcomes of patients with Takayasu arteritis (TAK).

**Methods:**

A retrospective cohort study including 81 TAK patients was carried out. Polymorphisms of the genes *HSD11B1* (rs11119328), *FKBP5* (rs1360780) and *NR3C1* (N363S, Bcll, TthIIII1, ER22/23EK and 9ß) were genotyped by the Sanger technique. Associations between the gene variants and the haplotypes (HT) of the *NR3C1* gene with variables related to the outcome of TAK and glucocorticoid (GC)-related adverse events (AEs) were analyzed.

**Results:**

The polymorphism 9β of the *NR3C1* gene, which leads to decreased GC sensitivity, was associated with a higher frequency of GC-related AEs [3.0 (2.0-3.8) vs. 2.0 (1.0-3.0); *p* = 0.002] and weight gain (37.5% vs. 8.9%; *p* = 0.012). Worsening glucose tolerance (i.e., a key GC-related AE) was an independent risk factor for acute ischemic events [odds ratio (OR) between 8.9 and 10.2] in all multivariate logistic regression models that included one of the polymorphisms in each model. Moreover, the carriage of 9β in the *NR3C1* gene was also an independent risk factor for developing ischemic arterial events (OR: 4.4, 95% confidence interval: 1.1-18.3). None of the other polymorphisms of *NR3C1*, *HSD11B1* and *FKBP5* were associated with TAK features or outcomes, nor with GC-related AEs.

**Conclusion:**

Worsening glucose tolerance and the carriage of 9β of the *NR3C1* gene were independent risk factors for acute ischemic events in TAK. The 9β polymorphism of the *NR3C1* gene was associated with GC-related AEs in TAK in our patient population. None of the gene variants were predictors of sustained remission or arterial progression.

## Highlights

- Worsening glucose tolerance and the carriage of 9β of the *NR3C1* gene were risk factors for acute ischemic events in TAK.
- Carriage of 9β of the NR3C1 gene was associated with a higher median of GC-related adverse events.
- Other polymorphisms of *NR3C1*, *FKBP5* and *HSD11B1* genes were not associated with outcomes in TAK.

## Introduction

Takayasu arteritis (TAK) is a chronic granulomatous systemic vasculitis, with no defined etiology, that affects large vessels, involving the aorta and its main branches, as well as pulmonary arteries (1). The inflammatory process begins in the adventitia and progresses to all layers of the artery, resulting in concentric thickening of the arterial wall that may lead to structural changes such as stenosis, occlusion, dilatation or aneurysm (2).

The treatment of TAK is mainly based on glucocorticoid (GC) use in association with conventional synthetic disease-modifying antirheumatic drugs (csDMARDs) or biological DMARDs (bDMARDs) (3–6). The long-term use of GC in TAK patients leads to an array of adverse events (AEs) that differ widely between patients and different strategies should be applied to minimize the burden of GC toxicity (7, 8).

Pharmacogenomics is an area of genetics that assesses the influence of individual genetic profiles on the effectiveness and AEs of therapeutic agents. It may be applied to the management of several diseases and is useful the individualization of treatment according to genetic variability in drug receptors or in enzymes involved in drug metabolism (9).

Tissue sensitivity to GC may be affected by different variables linked to pharmacogenomics. Firstly, the regulation of GC bioavailability may be influenced by the conversion of cortisone into cortisol, the active form of GC, by 11β hydroxysteroid dehydrogenase type 1 (11βHD1), an enzyme found mainly in the liver and the adipose tissue (10). Then, GC is bound to its receptors and its signaling pathways are activated as it exerts immunosuppressive, anti-inflammatory, and antiallergic effects on different immune cells through genomic and non-genomic mechanisms (11).

The polymorphism rs11119328 of the *HSD11B1* gene is an intronic variant (C>A/C>T) commonly found in the general population with a minor allele A frequency ranging between 0.186 and 0.236. This polymorphism reduces the expression of the 11βHD1 enzyme and results in lower bioavailability of the activated cortisol (12). In patients with antineutrophil cytoplasmic antibody (ANCA)-associated vasculitis, the carriage of rs11119328 was associated with an increased relapse rate (13).

The *FKBP5* gene is located in the short arm of chromosome 6 (6p21) and composed of 10 exons. To date, several intronic *FKBP5* polymorphisms have been described, especially rs1360780 (T>A/T>C) which is located in intron 2 (14). The T allele carriage leads to decreased tissue sensitivity to GC, and it is associated with psychiatric conditions including depression, borderline personality disorder, post-traumatic stress and suicidality (15). Indeed, decreased GC sensitivity is associated with psychiatric events (16).

Activated cortisone exerts its genomic effect by binding to GC receptors in the soluble and inactivated form in the cytoplasm of nucleated cells. The *NR3C1* gene encodes the glucocorticoid receptor (GR) and is located in the long arm of chromosome 5 (5q31.3) with 9 exons and 4.1kb (12, 13). The *NR3C1* gene is very polymorphic; some of its polymorphisms have a functional impact on the effects of cortisol and the pharmacodynamics of exogenous GC. Tissue sensitivity to GC is increased upon the carriage of rs56149945 (N363S) and rs41423247 (BclI) polymorphisms of the *NR3C1* gene, while the polymorphisms rs6189 (ER22/23EK), rs6190 (ER22/23EK) and rs6198 (GR-9β) lead to decreased GC sensitivity (17). Furthermore, rs10052957 (TthIII1), another polymorphism in the promoter region of the *NR3C1* gene, is characterized by the single base change of cytosine for a thymine (C>T) (18). TthIII1 alone does not impact GC sensitivity, but the concomitant carriage with ER22/23EK decreases GC sensitivity (19).

This study aims to evaluate different polymorphisms of the *NR3C1*, *FKBP5* and *HSD11B1* genes and their impact on the clinical outcomes of TAK and GC-related AEs.

## Patients and methods

### Study design and patients

We carried out a retrospective cohort study with prospective genetic analysis, including 81 TAK patients who were selected from the Vasculitis Outpatient Clinic of the Rheumatology Division at the Universidade Federal de São Paulo/Escola Paulista de Medicina (Unifesp/EPM) between March 2022 and August 2023. The inclusion criteria were age ≥ 18 years, fulfilment of the 2022 American College of Rheumatology/European Alliance of Associations for Rheumatology criteria for TAK (20), treatment with GC at any stage after diagnosis of the disease and written informed consent. Patients with chronic infectious diseases or systemic inflammatory/autoimmune diseases associated with TAK were excluded. The study protocol was approved by the Institutional Ethics Committee of Unifesp/EPM (CAAE: 45377121.5.0000.5505) and all study procedures were carried out according to the Declaration of Helsinki and its updates.

### Data collection

Patients’ information was collected from the medical records during their follow-up between the years 2014 and 2023. Details about clinical characteristics and outcome measures evaluated in the study are depicted in **Supplementary Table S1**. Relevant comorbidities for mortality risk were scored using the Charlson Comorbidity Index (21). Adverse events attributed to GC use were defined according to the Glucocorticoid Toxicity Index (GTI), as shown in **Supplementary Table S2**. The GC-related AEs included GC-induced hyperlipidemia, worsening systemic hypertension, worsening glucose tolerance, deterioration of bone mineral density (BMD), weight gain, cataract, skin lesions and severe infection. Worsening glucose tolerance included patients with previous diagnosis of diabetes, and those who developed hemoglobin A1c above 5.7% after GC therapy (22). Permanent damage related to TAK was assessed using two tools: the Vasculitis Damage Index (VDI) and the Takayasu Arteritis Damage Score (TADS) (23, 24). Stable disease in TAK was defined as the absence of disease relapse or of the development of new arterial lesions in previously unaffected vascular territories during follow-up (25).

### Genotyping and determination of haplotypes

**Figure 1:**
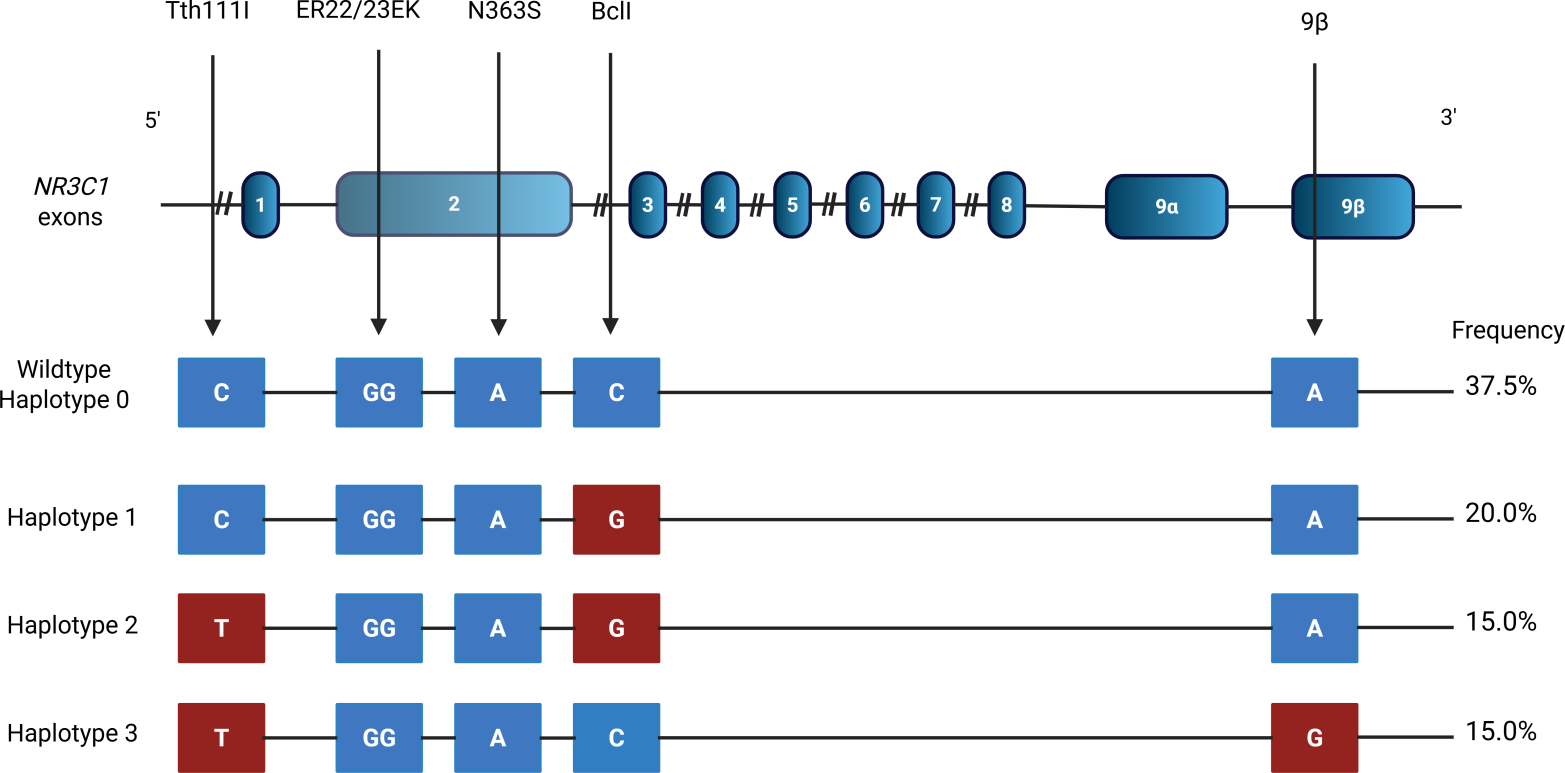
Description of the haplotypes of the NR3C1 gene and their respective frequencies. Nucleotide exchanges are indicated in red.

After DNA extraction from the peripheral blood, genotyping was carried out in the Genetics Laboratory at UNIFESP-EPM. Polymorphism genotyping was performed using the Sanger sequencing method. The following polymorphisms were evaluated TthIII1 (rs10052957), ER22/23EK (rs6189/6190), N363S (rs56149945) BclI (rs41423247) and 9β (rs6198) in the *NR3C1* gene; rs11119328 in the *HSD11B1* gene; and rs1360780 in the *FKBP5* gene. The haplotypes (HT) of the polymorphisms in the *NR3C1* gene were determined by the PHASE software as previously reported (13, 26). HT0 was defined as the absence of any of the above-mentioned polymorphisms, HT1 as the presence of the minor allele of BclI, HT2 as the presence of the minor allele of TthIII1 and BclI, and HT3 as the presence of the minor allele of TthIII1 and 9ß. Other *NR3C1* HT with a frequency below 5% were not analyzed. **Figure 1** depicts the location of the SNP of the *NR3C1* gene and all HT evaluated.

### Statistical analysis

Descriptive statistics included mean and standard deviation or median and interquartile range (IQR), for quantitative variables and frequency with proportion, for categorical variables. Continuous variables were compared for two groups using Student’s *t*-test for normally distributed variables or Mann-Whitney’s U test for variables with non-Gaussian distribution. Comparisons among three groups for continuous variables were performed using the Kruskal-Wallis test or by the one-way analysis of variance (ANOVA). The analysis of covariance (ANCOVA) was used to compare the cumulative prednisone dose adjusted for the duration of therapy between the carriers and non-carriers of each polymorphism and haplotypes. Categorical variables were evaluated using the chi-square test or Fisher’s exact test. Multivariate logistic regression models were built to evaluate predictors of acute arterial ischemic events and stable disease (i.e., no new arterial lesions during follow-up), and results were expressed as odds ratios (OR) with 95% confidence intervals (95% CI). The accepted significance level was 5% (*p*-value < 0.05). The IBM SPSS software for Windows version 21.0 (Armonk, NY) was used to perform statistical analysis of the data. GraphPad Prism for Windows version 9.0 (Boston, MA) was used to build graphs. The Hardy-Weinberg equilibrium assessment was performed using R Software version 4.3.2.

## Results

### Characteristics of TAK patients

**Figure 2:**
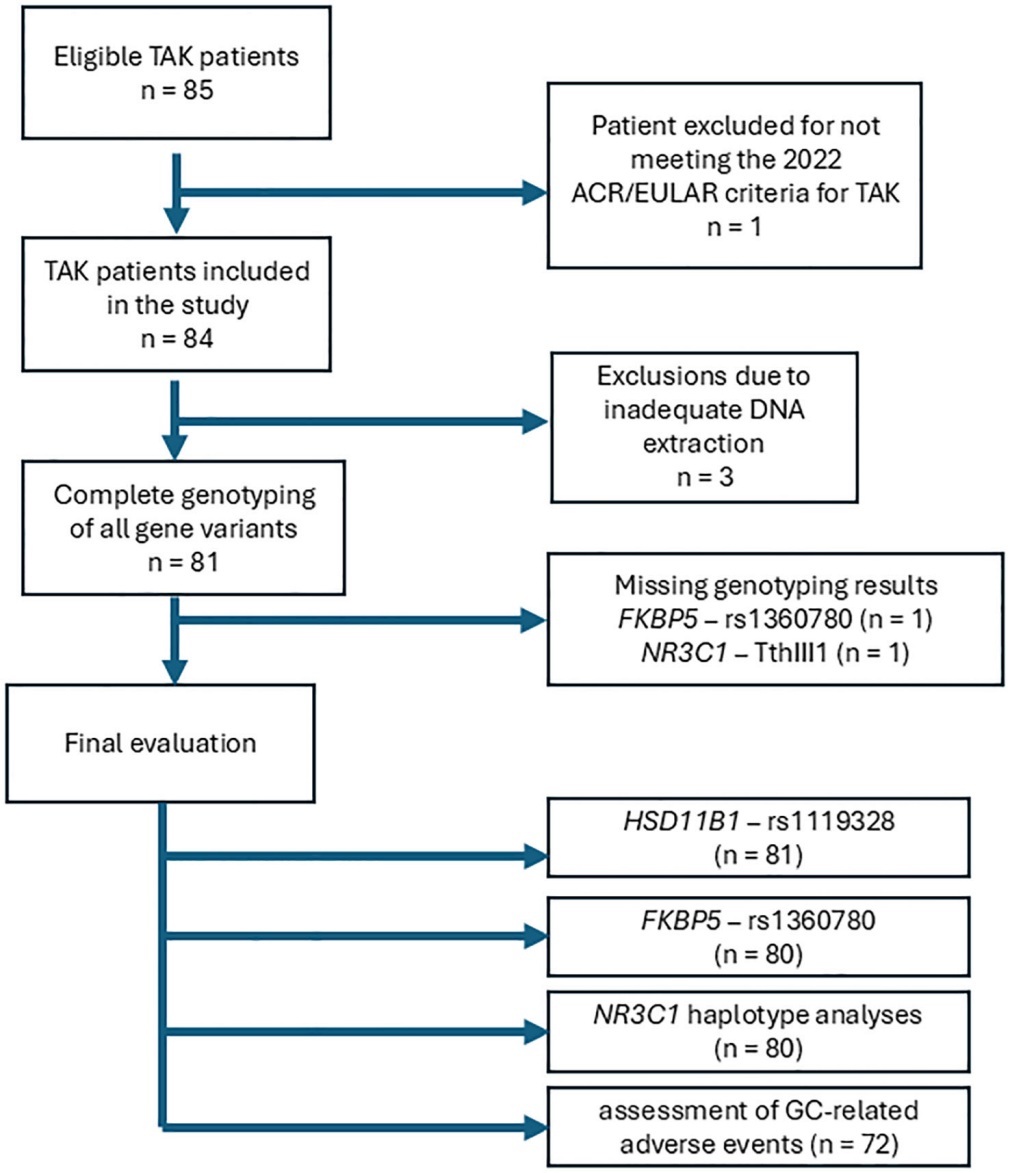
Flowchart of inclusion of patients in the study and analysis of the different polymorphisms and haplotypes.

Eighty-one TAK patients were included in the study. **Figure 2** describes patients’ inclusion in the study, as well as polymorphisms and haplotypes assessed in the study. The patients were mostly female (90.1%) and had a mean age of 29.6 ± 11.4 years, and a median of 85.0 months (54.0-103.0) of follow-up. Acute arterial ischemic events were observed in 28.4% of the patients and coronary artery disease was more common than stroke or transient ischemic attacks. **Table 1** describes the disease characteristics and ischemic events of TAK patients. Most patients were Whites or Mestizos (i.e., 84% of study participants). Angiographic type V was the most frequent type (71.6%), according to the classification of Hata and Numano (27), followed by types I (13.6%), IV (7.4%) and IIb (4.9%) (**Table 1**).

**Table 1:** Characteristics of TAK patients evaluated in the study.

Variables	Results (n = 81)
Age at diagnosis, years	29.6 ± 11.4
Race	
Whites, n (%)	46 (56.8)
Mestizos, n (%)	22 (27.2)
Blacks, n (%)	10 (12.3)
Asians, n (%)	3 (3.7)
Median disease duration, months	85.0 (54.0-103.0)
Acute phase reactants at diagnosis	
ESR, mm/hour	29.5 (14.0-50.3)
CRP, mg/L	4.9 (1.0-10.7)
Angiographic types	
Type V, n (%)	58 (71.6)
Type I, n (%)	11 (13.6)
Type IV, n (%)	6 (7.4)
Type IIb, n (%)	4 (4.9)
Type IIa, n (%)	1 (1.2)
Type III, n (%)	1 (1.2)
Ischemic events, n (%)	23 (28.4)
Number of ischemic events	32
MI, n (%)	7 (30.4)
Angina pectoris, n (%)	9 (39.1)
TIA, n (%)	4 (17.4)
Stroke, n (%)	6 (26.1)
Abdominal angina, n (%)	5 (21.7)
Subclavian steal syndrome, n (%)	13 (16.0)
Renovascular hypertension, n (%)	19 (23.5)
Involvement of pulmonary arteries, n (%)	14 (17.3)
Progression of arterial lesions, n (%)	42 (51.9)

TIA, Transient ischemic attack; MI, Myocardial infarction; n, Number of patients; ESR, Erythrocyte sedimentation rate; CRP, C-reactive protein.

Approximately half of the patients had progressive arterial lesions (i.e., the development of arterial lesions in previously unaffected territories) during the follow-up period (51.9%). TAK patients presented renovascular hypertension (23.5%), pulmonary artery involvement (17.3%) and subclavian steal syndrome (16.0%) (**Table 1**). At the end of the follow-up, the median and IQR were [2.0 (1.0-3.0)] for the Charlson Comorbidity Index, [2.0 (2.0-4.0)] for VDI and [5.0 (3.0-7.0)] for TADS.

Regarding TAK therapy with csDMARDs, methotrexate, leflunomide and azathioprine were used at any time during the follow-up in 73 (90.1%), 54 (66.6%) and 27 (33.3%) patients, respectively. On the other hand, bDMARDs, mostly TNF inhibitors, were prescribed for 55.6% of the patients.

### GC-related AEs

At the end of the follow-up, the median cumulative prednisone dose was 10,741.00 mg (5,840.63-22,547.88) and the median GTI score was 46.0 (19.0-76.0). At least one GC-related AE was observed in 82.7% of patients, and the median number of GC-related AEs was 2.0 (1.0-3.0). The frequencies of GC-related AEs were as follows: GC-induced hyperlipidemia (50.6%), worsening systemic hypertension (27.2%), worsening glucose tolerance (19.8%), deterioration of BMD (19.8%), weight gain (13.6%), severe infection (8.6%), skin lesions (6.2%), and cataract (3.7%). Among patients with worsening glucose tolerance, 81.3% had had a previous diagnosis of diabetes and needed to increase their diabetic medication, whereas 18.7% developed HbA1c levels above 5.7% upon GC therapy. Osteonecrosis of the femoral head and GC-induced psychosis were rare complications of GC therapy as they were each observed in only one TAK patient.

### Genotyping of TAK patients for the HSD11B1, FKBP5 and NR3C1 polymorphisms

The minor allele frequencies of the *HSD11B1* and *FKBP5* gene polymorphisms (i.e., rs11119328 and rs1360780) were 33.3% and 55.6%, respectively, while the minor alleles of *NR3C1* polymorphisms were found in 37.0% for BclI, 19.7% for 9β, 3.7% for ER22/23EK and 1.2% for N363S. The minor alleles were predominantly in heterozygosis. The minor allele of the TthIII1 polymorphism was present in 41.3% of TAK patients. **Supplementary Table S3** depicts the number of TAK patients who are homozygous and heterozygous for each polymorphism of *HSD11B1*, *FKBP5* and *NR3C1* genes. The rs11119328, rs1360780, BclI and 9β alleles were all in Hardy-Weinberg equilibrium. The frequencies of HT1, HT2 and HT3 of the *NR3C1* gene were 20%, 15% and 15% respectively. HT4 and HT5 were only found in 2 and 1 TAK patients, respectively.

The frequency of minor alleles of the *HSD11B*, *FKBP5* and *NR3C1* genes was compared among the ethnic groups (i.e., Whites, Mestizos and Blacks) with TAK. No significant differences were found in the carriage of the minor alleles of *HSD11B*, *FKBP5* and *NR3C1* genes among ethnic groups of TAK patients (**Supplementary Table S4**). These analyses did not include Asians since only three individuals had an Asian background in this study. Each of the rs1119382, rs1360780 and BclI polymorphisms was found in only one Asian patient. The 9β and TthIII1 polymorphisms of the *NR3C1* gene were not found in Asians.

### Associations between the carriage of HSD11B1, FKBP5 and NR3C1 polymorphisms and the outcomes of TAK

No significant associations were found between the carriage of the polymorphisms rs11119328 of *HSD11B1*, rs1360780 of *FKBP5*, and BclI and 9β of *NR3C1* and disease features or outcomes of TAK such as angiographic type V, subclavian steal syndrome, renovascular hypertension, pulmonary artery involvement, sustained remission, need for therapy with bDMARD, progressive arterial lesions, TADS score, VDI, and need for vascular interventions (**Tables 2**, **3**). Additionally, comparisons between carriers and non-carriers of *HSD11B1*, *FKBP5*, and BclI polymorphisms yielded no significant results in regard to GC-related AEs, severe infections, GTI score, CCI and cumulative prednisone dose (**Tables 2**, **3**). However, carriers of the 9β polymorphism had a higher median number of GC-related AEs compared to non-carriers (**Table 3**).

**Table 2:** Associations between TAK features, outcomes and GC-related variables with *HSD11B1* and *FKBP5* polymorphisms carriage.

Variables	Carriers (n = 27)	Non carriers (n = 54)	*p*	Carriers (n = 44)	Non carriers (n =35)	*p*
*HSD11B1* rs11119328				*FKBP5* rs1360780		
*TAK features and outcomes*				*TAK features and outcomes*		
Subclavian steal syndrome, n (%)	6 (22.2)	7 (13.0)	0.341	7 (15.9)	5 (14.3)	0.842
Renovascular hypertension, n (%)	7 (25.9)	12 (22.2)	0.711	12 (27.3)	7 (20.0)	0.452
Pulmonary artery involvement, n (%)	5 (18.5)	9 (16.7)	1.000	9 (20.5)	5 (14.3)	0.476
Angiographic type V, n (%)	20 (74.1)	38 (70.4)	0.727	32 (72.7)	25 (71.4)	0.898
Ischemic events, n (%)	6 (22.2)	17 (31.5)	0.384	11 (25.0)	11 (31.4)	0.527
Sustained remission, n (%)	16 (59.3)	33 (61.1)	0.872	25 (56.8)	22 (62.9)	0.587
Progression of arterial lesions, n (%)	15 (55.6)	27 (50.0)	0.637	21 (47.7)	21 (60.0)	0.278
Need for bDMARD therapy, n (%)	18 (66.7)	27 (50.0)	0.155	24 (54.5)	21 (60.0)	0.627
TADS	5.0 (2.5-8.0)	4.5 (3.0-6.7)	0.965	5.0 (2.3-6.0)	4.5 (3.0-8.8)	0.264
VDI	3.0 (1.2-4.0)	2.0 (2.0-3.0)	0.640	2.0 (2.0-3.0)	3.0 (2.0-5.0)	0.066
Vascular interventions, n (%)	9 (33.3)	18 (33.3)	1.000	15 (34.1)	12 (34.3)	0.986
*GC-related variables*				*GC-related variables*		
Presence of GC-related AEs, n (%)	16 (66.7)	32 (66.7)	1.000	28 (66.7)	20 (69.0)	0.839
Number of GC-related AEs, n (%)	1.5 (1.0-3.0)	2.0 (0.3-3.0)	0.912	2.0 (1.0-3.0)	3.0 (1.0-3.0)	0.378
Severe infections, n (%)	3 (12.5)	3 (6.3)	0.393	3 (7.1)	3 (10.3)	0.683
GTI	40.0 (19.0-91.5)	46.0 (19.0-71.0)	0.598	38.5 (19.0-74.3)	50.0 (19.0-92.0)	0.700
CCI	1.0 (1.0-2.5)	2.0 (1.0-3.0)	0.380	1.0 (1.0-3.0)	2.0 (1.0-3.0)	0.689
Cumulative prednisone dose, mg	14,553.75 ± 1,413.66	13,126.49 ± 995.78	0.414	13,334.54 ± 1,072.72	14,124.53 ± 1,291.86	0.640

AEs, Adverse events; bDMARD, Biological Disease-Modifying Antirheumatic Drug; CCI, Charlson Comorbidity Index; GC, Glucocorticoid; GTI, Glucocorticoid Toxicity Index; n, Number of patients; TADS, Takayasu arteritis Damage Score; TAK, Takayasu arteritis. The mean cumulative dose of prednisone was adjusted to the duration of therapy.

**Table 3:** Associations between TAK features, outcomes and GC-related variables with *NR3C1* polymorphisms carriage.

Variables	Carriers (n = 28)	Non carriers (n = 44)	*p*	Carriers (n = 16)	Non carriers (n = 56)	*p*
*NR3C1* BclI				*NR3C1* 9β		
*TAK features and outcomes*				*TAK features and outcomes*		
Subclavian steal syndrome, n (%)	6 (20.0)	7 (13.72)	0.536	5 (31.3)	8 (12.3)	0.120
Renovascular hypertension, n (%)	10 (33.3)	9 (17.6)	0.108	3 (18.8)	16 (24.6)	0.751
Pulmonary artery involvement, n (%)	3 (10.0)	11 (21.6)	0.184	4 (25.0)	10 (15.4)	0.460
Angiographic type V, n (%)	23 (76.7)	35 (68.6)	0.438	10 (62.5)	48 (73.8)	0.371
Ischemic events, n (%)	9 (30.0)	14 (27.5)	0.806	8 (50.0)	15 (23.1)	0.060
Sustained remission, n (%)	17 (56.7)	32 (62.7)	0.589	7 (43.8)	42(64.6)	0.126
Progression of arterial lesions, n (%)	15 (50.0)	27 (52.9)	0.798	8 (50.0)	34 (52.3)	0.869
Need for bDMARD therapy, n (%)	16 (53.3)	29 (56.9)	0.758	9 (56.3)	36 (55.4)	0.950
TADS	4.0 (3.0-6.7)	5.0 (3.5-7.5)	0.263	6.0 (4.3-9.0)	4.0 (3.0-6.0)	0.117
VDI	2.0 (1.0-3.2)	3.0 (2.0-4.0)	0.416	3.0 (2.0-4.5)	2.0 (2.0-4.0)	0.381
Vascular interventions, n (%)	10 (33.3)	17 (33.3)	1.000	5 (31.3)	22 (33.8)	0.844
*GC-related variables*				*GC-related variables*		
Presence of GC-related AEs, n (%)	18 (64.3)	30 (68.2)	0.732	13 (81.3)	35 (62.5)	0.161
Number of GC-related AEs, n (%)	2.0 (1.0-3.0)	1.0 (1.0-3.0)	0.272	3.0 (2.0-3.8)	2.0 (1.0-3.0)	0.002*
Severe infections, n (%)	3 (10.7)	3 (6.8)	0.672	2 (12.5)	4 (7.1)	0.609
GTI	29.5 (10.0-80.5)	49.0 (19.0-75.0)	0.420	61.0 (20.5-84.5)	30.0 (19.0-74.0)	0.289
CCI	1.5 (1.0-3.8)	1.0 (1.0-3.0)	0.773	2.0 (1.0-3.8)	1.0 (1.0-3.0)	0.386
Cumulative prednisone dose, mg	13,887.03 ± 1,304.93	13,421.02 ± 1,040.81	0.781	14,594.46 ± 1,273.43	12,933.08 ± 1,045.68	0.902

AEs, Adverse events; bDMARD, Biological Disease-Modifying Antirheumatic Drug; CCI, Charlson Comorbidity Index; GC, Glucocorticoid; GTI, Glucocorticoid Toxicity Index; n, Number of patients; TADS, Takayasu arteritis Damage Score; TAK, Takayasu arteritis; *, Flags significant results. The mean cumulative dose of prednisone was adjusted to the duration of therapy.

For specific GC-related AEs as defined by the GTI, such as worsening lipid profile, exacerbation of hypertension, worsening glucose tolerance, deterioration of BMD, weight gain, cataracts, and skin lesions, only 9β carriers had a higher frequency of weight gain than non-carriers (37.5% vs. 8.9%; *p* = 0.012) (**Supplementary Table S5**). No other significant differences were found concerning the carriage of other polymorphisms (i.e., rs11119328 of *HSD11B1*, rs1360780 of *FKBP5* and BclI of *NR3C1*) and specific GC-related AEs (**Supplementary Tables S5**, **S6**).

Due to the low frequencies of ER22/23EK and N363S polymorphisms among TAK patients, no further analyses were performed for TAK features, outcomes and GC-related AEs.

### NR3C1 gene haplotypes in TAK patients

**Table 4** compares the outcomes of TAK and GC-related AEs among TAK patients carrying HT1, HT2, and HT3 of the *NR3C1* gene. Despite significant differences in the median number of GC-related AEs among HTs (*p* = 0.032), we only found a tendency for a higher median number of GC-related AEs in carriers of HT3 compared to the carriers of HT1 and HT2 (*p* = 0.025 and *p* = 0.020; respectively) (Bonferroni’s correction – *p* = 0.016) in the *post-hoc* analyses. For specific GC-related AEs (**Supplementary Table S7**), only a tendency for a higher frequency of weight gain was observed in HT3 compared to HT1 carriers (33.3% vs. 0.0%; *p* = 0.028) in the *post-hoc* analysis. No differences were found between HT2 and HT3 carriers regarding weight gain (9.1% vs. 33.3%; *p* > 0.05). No other significant differences were observed among HT1, HT2 and HT3 carriers (**Table 4**, **Supplementary Table S7**).

**Table 4:** Comparisons among patients carrying different *NR3C1* haplotypes.

Variables	HT1 (n=16)	HT2 (n=12)	HT3 (n=12)	*p*
TAK features and outcomes				
Subclavian steal syndrome, n (%)	3 (18.8)	2 (16.7)	3 (25.0)	0.867
Renovascular hypertension, n (%)	6 (37.5)	4 (33.3)	3 (25.0)	0.781
Pulmonary artery involvement, n (%)	2 (12.5)	1 (8.3)	4 (33.3)	0.217
Angiographic type V, n (%)	14 (87.5)	9 (75.0)	9 (75.0)	0.626
Ischemic events, n (%)	5 (31.3)	2 (16.7)	4 (33.3)	0.599
Sustained remission, n (%)	8 (50.0)	9 (75.0)	6 (50.0)	0.342
Progression of arterial lesions, n (%)	8 (50.0)	6 (50.0)	6 (50.0)	1.000
Need for bDMARD therapy, n (%)	8 (50.0)	6 (50.0)	6 (50.0)	1.000
TADS	3.5 (2.8-6.0)	4.0 (3.0-6.8)	5.0 (2.5-9.0)	0.586
GC-related variables				
GC-related adverse events, n (%)	9 (60.0)	7 (63.6)	9 (75.0)	0.705
Number of adverse events	1.5 (1.0-2.8)	1.5 (1.0-2.3)	3.0 (2.0-3.0)	0.032*
Severe infections, n (%)	3 (20.0)	0 (0.0)	2 (16.7)	0.300
GTI Score	82.5 (16.8-99.5)	24.0 (10.0-48.0)	50.0 (20.5-89.0)	0.119
CCI Score	1.0 (1.0-2.0)	2.0 (1.0-4.0)	1.5 (1.0-2.8)	0.711
Cumulative dose of prednisone, mg	13,527.24 ± 1,972.50	15,556.91 ± 2,314.90	15,325.06 ± 2,201.36	0.754

bDMARD, Biological Disease-Modifying Antirheumatic Drug; CCI, Charlson Comorbidity Index; GC, Glucocorticoid; GTI, Glucocorticoid Toxicity Index; HT, Haplotype; n, number of patients; TADS, Takayasu arteritis Damage Score; *, Flags significant results. The mean cumulative dose of prednisone was adjusted to the duration of therapy.

### Predictors of ischemic arterial events in patients with TAK

We built multivariate logistic regression models to analyze predictors of acute ischemic events and stable disease (i.e., no disease relapses or progression of arterial lesions) in TAK. In the regression models, we evaluated one of the following polymorphisms: rs11119328 of *HSD11B1*, rs1360780 of *FKBP5* and BclI of *NR3C1* gene with other relevant independent variables.

In the regression models for acute ischemic events, we included sustained remission, bDMARD use, vascular interventions, worsening glucose tolerance, GC-induced hyperlipidemia, worsening systemic hypertension, progression of arterial lesions and each polymorphism as independent variables. In all the regression models, worsening glucose tolerance was an independent risk factor for acute ischemic events in TAK. However, in the regression model with the 9β polymorphism of *NR3C1*, both worsening glucose tolerance and 9β carriage were predictors of acute ischemic events in TAK (**Table 5**).

**Table 5:** Logistic regression models to evaluate predictors for acute ischemic events in TAK.

Variables	OR	95% CI	*p*
*HSD11B1* rs11119328 model			
rs1119328	0.685	0.204-2.307	0.542
Sustained remission	0.656	0.180-2.398	0.524
bDMARDs	2.085	0.652-6.671	0.215
Vascular interventions	1.102	0.292-4.163	0.886
Worsening glucose tolerance	8.928	2.132-37.391	0.003*
GC-induced hyperlipidemia	0.595	0.174-2.032	0.407
Worsening hypertension	0.543	0.165-1.785	0.314
Progression of arterial lesions	0.518	0.155-1.733	0.286
*FKBP5* rs1360780 model			
rs1360780	0.785	0.250-2.465	0.679
Sustained remission	0.565	0.149-2.140	0.401
bDMARDs	2.184	0.666-7.164	0.198
Vascular interventions	1.251	0.318-4.931	0.749
Worsening glucose tolerance	9.503	2.220-40.676	0.002*
GC-induced hyperlipidemia	0.643	0.181-2.287	0.495
Worsening hypertension	0.583	0.177-1.924	0.376
Progression of arterial lesions	0.504	0.146-1.745	0.280
*NR3C1* BclI			
BclI	1.014	0.325-3.164	0.981
Sustained remission	0.639	0.174-2.342	0.499
bDMARDs	1.974	0.627-6.216	0.245
Vascular interventions	1.132	0.296-4.327	0.856
Worsening glucose tolerance	9.287	2.223-38.793	0.002*
GC-induced hyperlipidemia	0.585	0.167-2.054	0.403
Worsening hypertension	0.545	0.167-1.775	0.313
Progression of arterial lesions	0.500	0.149-1.675	0.261
NR3C1 9β			
9β	4.371	1.042-18.341	0.044*
Sustained remission	0.863	0.225-3.314	0.830
bDMARDs	2.102	0.636-6.952	0.223
Vascular interventions	1.335	0.343-5.201	0.677
Worsening glucose tolerance	10.227	2.295-45.581	0.002*
GC-induced hyperlipidemia	0.354	0.085-1.474	0.354
Worsening hypertension	0.618	0.179-2.139	0.448
Progression of arterial lesions	0.622	0.177-2.191	0.448

95% CI, 95% Confidence interval; bDMARD, Biological Disease-Modifying Antirheumatic Drug; GC, Glucocorticoid; OR, Odds ratio; *, Flags significant results.

In the models analyzing predictors for stable disease in TAK, we added methylprednisolone use, bDMARDs, sustained remission, vascular interventions, acute ischemic events, angiographic type V and each polymorphism as independent variables. In these regression models, no polymorphisms were predictors of stable disease. In all but one regression model, sustained remission was associated with stable disease in TAK, and in one of the regression models, the use of bDMARDs was inversely associated with stable disease (**Table 6**).

**Table 6:** Logistic regression models to evaluate predictors for stable disease in TAK.

Variables	OR	95% CI	*p*
*HSD11B1* rs11119328 model			
rs1119328	0.899	0.281-2.878	0.858
IV methylprednisolone pulse therapy	2.894	0.697-12.021	0.144
bDMARDs	0.274	0.070-1.068	0.062
Sustained remission	3.395	1.006-11.458	0.049*
Vascular interventions	1.038	0.305-3.527	0.953
Acute ischemic events	1.342	0.410-4.394	0.626
Angiographic type V	0.629	0.188-2.101	0.451
*FKBP5* rs1360780 model			
rs1360780	1.637	0.534-5.021	0.389
IV methylprednisolone pulse therapy	2.934	0.718-11.993	0.134
bDMARDs	0.304	0.079-1.172	0.084
Sustained remission	3.256	0.938-11.306	0.063
Vascular interventions	1.037	0.291-3.690	0.956
Acute ischemic events	1.320	0.379-4.594	0.663
Angiographic type V	0.568	0.167 -1.928	0.364
*NR3C1* BclI			
BclI	1.688	0.541-5.265	0.367
IV methylprednisolone pulse therapy	3.130	0.762-12.857	0.113
bDMARDs	0.257	0.068-0.975	0.046*
Sustained remission	3.611	1.057-12.332	0.040*
Vascular interventions	1.012	0.294-3.475	0.985
Acute ischemic events	1.319	0.400-4.346	0.649
Angiographic type V	0.590	0.174-1.999	0.397
NR3C1 9β			
9β	1.213	0.319-4.606	0.777
IV methylprednisolone pulse therapy	2.906	0.707-11.939	0.139
bDMARDs	0.270	0.071-1.024	0.054
Sustained remission	3.486	1.007-12.067	0.049*
Vascular interventions	1.034	0.303-3.528	0.957
Acute ischemic events	1.282	0.374-4.393	0.693
Angiographic type V	0.645	0.190-2.192	0.483

95% CI, 95% Confidence interval; bDMARD, Biological Disease-Modifying Antirheumatic Drug; GC, Glucocorticoid; OR, Odds ratio; *, Flags significant results.

## Discussion

In this study, the carriage of 9β and worsening glucose tolerance were independent risk factors for acute arterial ischemic events in TAK. In addition, 9β carriers were associated with a higher median number of GC-related AEs compared to non-carriers. No significant associations were found between the “other” polymorphisms of *NR3C1*, *FKBP5*, and *HSD11B1* genes and TAK features or outcomes. None of the polymorphisms of *NR3C1*, *FKBP5*, and *HSD11B1* genes, besides 9β, were associated with GC-related AEs. No significant associations were found between *NR3C1* HTs and TAK features, outcomes and GC-related AEs. None of the polymorphisms of *NR3C1*, *FKBP5*, and *HSD11B1* were predictors of stable disease (i.e., no angiographic progression).

This is the first study to evaluate the frequencies of the polymorphisms of the *NR3C1*, *FKBP5*, and *HSD11B1* genes in TAK and their associations with TAK features, outcomes, and GC-related AEs. We assessed whether, from a genetic point of view, GC sensitivity had any impact on TAK outcomes. Patients presenting decreased GC sensitivity required higher daily prednisone doses than usual to control disease activity, resulting in a higher burden of GC-related AEs, whereas patients presenting increased GC sensitivity required lower daily prednisone doses to control disease activity, possibly leading to a lower risk of developing AEs (28).

TAK therapy is based on long-term use of GC, resulting in high cumulative GC doses and the development of GC-related AEs (29). Furthermore, acute ischemic arterial events, mainly involving the cerebral and coronary territories, are common complications of TAK (30). Therefore, the evaluation of polymorphisms in the *NR3C1*, *FKBP5* and *HSD11B1* genes and their associations with clinical outcomes of TAK patients is of paramount importance for clinical practice. The carriage of specific polymorphisms indicates susceptibility to increased GC toxicity or a favorable therapeutic response.

The 9β polymorphism is associated with decreased GC sensitivity (31), which in theory may be related to poor control of disease activity upon GC therapy and hypothetically may lead to an increased risk of progression of arterial lesions and development of ischemic events. Therefore, the need for longer and higher GC doses to control disease activity may result in a higher frequency of GC-related AEs. This assumption aligns with the findings of our cohort, as 9β carriers had a higher median number of AEs and more frequent weight gain. Furthermore, 9β carriage was an independent risk factor for developing acute ischemic arterial events in patients with TAK, regardless of the risk factors for coronary artery disease.

In the literature, carriage of the 9β variant has been reported to be associated with systemic inflammatory conditions. The 9β variant has been shown to increase the chance of developing rheumatoid arthritis (32). In ANCA-associated vasculitis, this *NR3C1* variant is associated with an increased mortality rate and a higher chance of end-stage renal disease (13). This evidence points to poor control of the inflammatory process associated with the carriage of the 9β polymorphism, despite therapy. In addition, TAK patients present metabolic syndrome more frequently than healthy controls and the excessive number of risk factors for cardiovascular disease may be a confounding factor when analyzing the influence of 9β on acute arterial ischemic events (33, 34). Nonetheless, the carriage of 9β polymorphism and worsening glucose tolerance were independent predictors of this severe complication in our study. It has been reported that HT3 of the *NR3C1* gene, which contains the 9β polymorphism, is related to increased cardiovascular risk with increased inflammatory cytokines and carotid intima-media thickening in the general population (35). Conversely, a study of a multiethnic population in the Netherlands did not find any associations between 9β carriage and the age of onset of diabetes or metabolic parameters (36).

The *NR3C1* polymorphisms N363S, BclI, and 9β and their relationships with disease activity parameters were evaluated in patients with rheumatoid arthritis. The carriage of the minor allele of *Bcl*I or N363S (i.e., both increase GC sensitivity) was associated with lower baseline levels of disease activity (37). Conversely, the carriage of BclI increased the frequency of central nervous system manifestations, especially psychiatric symptoms, in patients with systemic lupus erythematosus (38).

Since the *NR3C1* gene is very polymorphic, it is worth mentioning that the carriage of polymorphisms with antagonist functions may result in nullified effects on GC sensitivity. Therefore, haplotypic analysis of the *NR3C1* gene may be more relevant compared to the analyses of individual polymorphisms. In our study, we evaluated only three haplotypes of the *NR3C1* gene (i.e., HT1, HT2 and HT3) since the frequencies of the other HTs, such as HT4 and HT5, were too low for further analyses. HT3 carriage encompasses the combination of the polymorphisms 9ß and TthIII1 in the *NR3C1* gene. The carriage of HT3 in TAK patients led to a trend for more frequent GC-related AEs compared to the carriage of other HTs, and a trend for a higher frequency of weight gain than HT1 carriage. The similarity of findings in the analysis of HT3 and the 9ß polymorphism indicates that the intronic variant Tthlll1 had little effect on the GC-decreased sensitivity exerted by 9ß. Indeed, the carriage of the polymorphism 9β was an independent risk factor for acute ischemic events in TAK patients. In all logistic regression models evaluating predictors of ischemic events in TAK, worsening glucose tolerance, as described by the GTI, was an independent risk factor for acute ischemic arterial events. This is in line with the evidence in the literature that diabetes is a major risk factor for acute ischemic events in the general population (39).

The variants BclI and N363S in the *NR3C1* gene are associated with increased sensitivity to GC, whereas 9β and ER22/23EK polymorphisms are associated with a decreased GC sensitivity (40). In this study, the most frequent polymorphisms of the *NR3C1* gene among TAK patients were BclI and 9β, which have antagonist effects regarding GC sensitivity. This is in line with previous reports in the literature, where the frequencies of the minor alleles in these polymorphisms in the dbSNP database vary from 16 to 36% and from 1 to 24%, respectively (41, 42). On the other hand, the rare *NR3C1* polymorphisms, described in 0-3% of individuals in databases that include data on Brazil, were infrequent among TAK patients, since only 3 individuals were carriers of the ER22/23EK polymorphism and only one patient had the N363S polymorphism (43, 44). The frequency of the minor allele in the intronic variant TthIII1 was 41.3% in TAK patients. This finding is consistent with the wide range of frequencies (1.9-60.7%) reported for different populations (45).

The minor allele of the rs11119328 polymorphism in the *HSD11B1* gene is associated with a decreased expression of the 11βHD1 enzyme and lower conversion of inactive cortisone into active cortisol. Theoretically, rs11119328 polymorphism, which has been studied in a few disorders, induces poor disease control in patients with systemic inflammatory diseases, and it has been studied in a few disorders. In ANCA-associated vasculitis, the carriage of rs11119328 minor allele was associated with a higher risk of disease relapse (13). Furthermore, kidney transplant patients presenting chronic rejection had upregulation of the cortisol-activating *HSD11B1* gene, indicating an unmet cortisol demand in chronic kidney transplant rejection. Conversely, lower 11βHD1 activity after discontinuing infliximab use in Crohn’s disease, increased the risk of mid/long-term clinical relapse (46, 47). In our study, the carriage of the rs11119328 A allele had no impact on disease features and outcomes of patients with TAK, as well as on GC-related AEs.

*FKBP5* is a polymorphic gene that encodes the protein FKBP5, a co-chaperone protein of the GC receptor (48). Most studies evaluating genetic variants of *FKBP5* focused mainly on its relations with psychiatric diseases. The *FKBP5* polymorphism of interest in our study was rs1360780, and even though it was found at a high frequency (55.7%) in TAK patients, no significant associations were found with TAK features and outcomes, as well as with GC-related AEs (14, 49). In a related study, no association was observed between rs1360780 and clinical outcomes in idiopathic thrombocytopenic purpura (50). However, rs1360780 carriage has already been described as a risk factor for bronchial asthma (51). No studies, besides ours, have described associations between rs1360780 carriage and outcomes in systemic inflammatory diseases.

Our study has its strengths and limitations. The main strengths are the long-term follow-up of TAK patients and the use of the Sanger technique, which increases precision in detecting genetic variants, for genotyping the polymorphisms of interest. The main limitations of the study are its single-center retrospective design, which is subject to recall bias, and the relatively small number of TAK patients included which limit generalizability of the results. The latter may be due to the rarity of the disease in Brazil (52). Additionally, limitations of this study include the absence of a control group and body mass index data of the participants.

In conclusion, 9β was the only polymorphism of the *NR3C1* gene to present significant associations in TAK in this cohort, as its carriage led to a higher frequency of GC-related AEs and weight gain. Worsening glucose tolerance and 9β in the *NR3C1* gene carriage were independent risk factors for acute ischemic events in TAK in our patient population. None of the other variants of the *NR3C1*, *HSD11B1* and *FKBP5* gene were associated with clinical outcomes or GC-related AEs in TAK.

## Data Availability

The data supporting the results of this study will be made available by the corresponding author upon reasonable request.
