# Supplementary material for: Gene variants of glucocorticoid activation pathways and the outcomes of patients with Takayasu arteritis – a retrospective cohort study
Source: Front Immunol. 2025 Oct 2;16:1675026. doi: 10.3389/fimmu.2025.1675026 (PMC12528102; doi:10.3389/fimmu.2025.1675026)
Supplement: Supplementary file 1 [file Table1.docx]

**Supplementary Tables**

**Supplementary Table S1 – Main outcomes evaluated in the study in relation to SNPs of the *HDS11B1*, *FKBP5* and *NR3C1 genes*.**

| **Variables** | |
| --- | --- |
| *Features and outcomes of TAK patients* | Subclavian steal syndrome |
|  | Renovascular hypertension |
|  | Involvement of pulmonary arteries |
|  | Angiographic type V |
|  | Ischemic events |
|  | Sustained remission |
|  | Need for intravenous pulse therapy with MTP |
|  | Need for the use of bDMARDs |
|  | Progression of arteriographic lesions |
|  | TADS score |
|  | VDI score |
|  | Vascular interventions |
| *GC-related variables* | Presence of adverse events from GCs |
|  | Number of adverse events |
|  | GC-induced hyperlipidemia |
|  | Worsening systemic hypertension |
|  | Worsening glucose tolerance |
|  | Deterioration of BMD |
|  | Weight gain |
|  | Cataract |
|  | Skin lesions |
|  | Severe infections |
|  | Cumulative dose of prednisone |
|  | GTI Score |
|  | CCI Score |

bDMARDs – Biological Disease-Modifying Antirheumatic Agents; BMD – Bone mass density; CCI – Charlson Comorbidity Index; GC – Glucocorticoid; GTI – Glucocorticoid Toxicity Index; TADS – Takaysu Arteritis Damage Score; VDI – Vasculitis Damage Index; MTP – Methylprednisolone; SNP – single nucleotide polymorphisms.

**Supplementary Table S2 – Definition of glucocorticoid-related adverse events adapted from the GTI.**

| **GC-related AEs** | **Definitions** |
| --- | --- |
| GC-induced hyperlipidemia | Worsening LDL > 10% to above target range or the need to increase in medication or an increase in LDL levels of > 10% despite treatment. |
| Worsening systemic hypertension | Increase in BP > 10% with systolic BP exceeding 120 mmHg or diastolic BP exceeding 85 mmHg with or without changes in medication or the need for an increase in anti-hypertensive medication. |
| Worsening glucose tolerance | HbA1c > 5.7% and increase to > 10% of baseline or the increase in diabetic medication even in the absence of 10% increase in HbA1c. |
| Deterioration of BMD | Decrease in BMD by 3%. |
| Weight gain | Increase in BMI of at least 2 BMI units to above the upper limit of normal BMI (24.9kg/m^2^). |
| Cataract | Cataract detected by an ophthalmologist. |
| Skin lesions | Acneiform rash, easy bruising, hirsutism, atrophy/striae, erosion, tears or ulcerations. |
| Severe infection | Grade 3 infections or complicated herpes zoster. |

BMD – Bone mineral density; BMI – Body mass index; BP – Blood pressure; GC – Glucocorticoid; LDL – Low density lipoprotein.

Supplementary Table S3 – Minor alleles of the polymorphisms in the *HSD11B1*, *FKBP5* and *NR3C1* genes.

| **Polymorphisms** | **Wild-type** | **Minor allele in heterozygosity** | **Minor allele in homozygosity** |
| --- | --- | --- | --- |
| *HSD11B1* |  |  |  |
| rs11119328 | CC (n=54) | CA (n=26) | AA (n=1) |
| *FKBP5* |  |  |  |
| rs1360780 | CC (n=35) | CT(n=36) | TT (n=8) |
| *NR3C1* |  |  |  |
| BclI (rs41423247) | CC (n=51) | CG (n=25) | GG (n=5) |
| 9β (rs6198) | AA (n=65) | GA (n=14) | GG (n=2) |
| ER22/23EK (rs6189/rs6190) | GG/GG (n=78) | GA/GA (n=2)  GA/GG (n=1) | AA (n=0) |
| N363S (rs56149945) | AA (n=80) | AG (n=1) | GG (n=0) |
| TthIII1 (rs10052957) | CC (n=47) | CT (=27) | TT (n=6) |

A – Adenine; C – Cytosine; G – Guanine; T – Thymine; n – Number of patients. Results are presented as the absolute number of genotyped patients for each polymorphism.

**Supplementary Table S4 – Minor alleles of the polymorphisms in the *HSD11B1*, *FKBP5* and *NR3C1* genes and ethnicity.**

| **Polymorphisms** | **Whites**  **(n = 46)** | **Mestizos**  **(n = 22)** | **Blacks**  **(n = 10)** | ***p*** |
| --- | --- | --- | --- | --- |
| rs11119382 of *HSD11B1*, n (%) | 13 (28.3) | 15 (68.2) | 5 (50.0) | 0.410 |
| rs1360780 of *FKBP5*, n (%) | 27 (58.7) | 9 (45.0) | 7 (70.0) | 0.385 |
| 9β of *NR3C1*, n (%) | 8 (17.4) | 5 (22.7) | 3 (30.0) | 0.640 |
| BclI of *NR3C1*, n (%) | 20 (43.5) | 6 (27.3) | 3 (30.0) | 0.382 |
| Tth111I of *NR3C1*, n (%) | 20 (43.5) | 10 (47.6) | 3 (30.0) | 0.645 |

n – Number of participants.

**Supplementary Table S5 – Carriage of *NR3C1* polymorphisms and GC-related adverse events.**

| **Variables** | **Carriers**  **(n = 28)** | **Non carriers**  **(n = 44)** | ***p*** | **Carriers**  **(n = 16)** | **Non carriers**  **(n = 56)** | ***p*** |
| --- | --- | --- | --- | --- | --- | --- |
| *NR3C1* BclI | | | | *NR3C1* 9β | | |
| GC-induced hyperlipidemia, n (%) | 12 (42.9) | 28 (63.6) | 0.084 | 12 (75.0) | 28 (50.0) | 0.076 |
| Worsening systemic hypertension, n (%) | 8 (28.6) | 16 (36.4) | 0.494 | 5 (31.3) | 19 (33.9) | 0.841 |
| Worsening glucose tolerance, n (%) | 6 (21.4) | 9 (20.5) | 0.921 | 5 (31.3) | 10 (17.9) | 0.299 |
| Deterioration of BMD, n (%) | 6 (21.4) | 8 (18.2) | 0.734 | 4 (25.0) | 10 (17.9) | 0.497 |
| Weight gain, n (%) | 2 (7.1) | 9 (20. 5) | 0.183 | 6 (37.5) | 5 (8.9) | 0.012* |
| Cataract, n (%) | 1 (3.6) | 2 (4.5) | 1.000 | 1 (6.3) | 2 (3.6) | 0.535 |
| Skin lesions, n (%) | 4 (14.3) | 1 (2.3) | 0.071 | 2 (12.5) | 3 (5.4) | 0.307 |
| ≥3 GC-related AEs, n (%) | 2 (7.1) | 2 (4.5) | 0.640 | 2 (12.5) | 2 (3.6) | 0.212 |

AEs – Adverse events; BMD – Bone mineral density; GC – Glucocorticoid; n – Number of patients.

**Supplementary Table S6 – Carriage of *HSD11B1* and *FKBP5* polymorphisms and GC-related adverse events.**

| **Variables** | **Carriers**  **(n = 27)** | **Non carriers**  **(n = 54)** | ***p*** | **Carriers**  **(n = 44)** | **Non carriers**  **(n =35)** | ***p*** |
| --- | --- | --- | --- | --- | --- | --- |
| *HSD11B1* rs11119382 | | | | *FKBP5* rs1360780 | | |
| GC-induced hyperlipidemia, n (%) | 13 (54.2) | 29 (60.4) | 0.612 | 22 (52.4) | 18 (62.1) | 0.418 |
| Worsening systemic hypertension, n (%) | 9 (37.5) | 15 (31.3) | 0.596 | 16 (38.1) | 8 (27.6) | 0.357 |
| Worsening glucose tolerance, n (%) | 4 (16.7) | 11 (22.9) | 0.538 | 8 (19.0) | 7 (24.1) | 0.606 |
| Deterioration of BMD, n (%) | 4 (16.7) | 10 (20.8) | 0.761 | 8 (19.0) | 6 (20.7) | 0.864 |
| Weight gain, n (%) | 3 (12.5) | 8 (16.7) | 0.741 | 7 (16.7) | 4 (13.8) | 1.000 |
| Cataract, n (%) | 0 (0.0) | 3 (6.3) | 0.546 | 1 (2.4) | 2 (6.9) | 0.563 |
| Skin lesions, n (%) | 1 (4.2) | 4 (8.3) | 0.659 | 2 (4.8) | 3 (10.3) | 0.393 |
| ≥3 GC-related AEs, n (%) | 0 (0.0) | 4 (8.3) | 0.294 | 3 (7.1) | 1 (3.4) | 0.640 |

AEs – Adverse events; BMD – Bone mineral density; GC – Glucocorticoid; n – Number of patients.

**Supplementary Table S7 – Haplotypes of *NR3C1* polymorphisms and GC-related adverse events.**

| **GC-related AEs** | **HT1**  **(n = 16)** | **HT2**  **(n = 12)** | **HT3**  **(n = 12)** | ***p*** |
| --- | --- | --- | --- | --- |
| GC-induced hyperlipidemia, n (%) | 6 (40.0) | 5 (45.5) | 9 (75.0) | 0.166 |
| Worsening systemic hypertension, n (%) | 4 (26.7) | 2 (18.2) | 5 (41.7) | 0.449 |
| Worsening glucose tolerance, n (%) | 4 (26.7) | 2 (18.2) | 3 (25.0) | 0.874 |
| Deterioration of BMD, n (%) | 3 (20.0) | 3 (27.3) | 2 (16.7) | 0.817 |
| Weight gain, n (%) | 0 (0.0) | 1 (9.1) | 4 (33.3) | 0.035* |
| Cataracts, n (%) | 0 (0.0) | 1 (9.1) | 0 (0.0) | 0.284 |
| Cutaneous lesions, n (%) | 1 (6.7) | 2 (18.2) | 1 (8.3) | 0.612 |
| ≥3 GC-induced AEs, n (%) | 0 (0.0) | 1 (9.1) | 3 (25.0) | 0.108 |

AEs – Adverse events; BMD – Bone mineral density; GC – Glucocorticoid; HT – Haplotypes; n – Number of patients; * - Flags significant results.
